# Semi-automated Modular Program Constructor for physiological modeling: Building cell and organ models

**DOI:** 10.12688/f1000research.7476.3

**Published:** 2016-06-16

**Authors:** Bartholomew Jardine, Gary M. Raymond, James B. Bassingthwaighte

**Affiliations:** 1 Department of Bioengineering, University of Washington, Seattle, WA, 98195, USA

**Keywords:** multi-scale modeling, JSim, systems biology, physiological modeling, reproducibility, uncertainty quantification

## Abstract

The Modular Program Constructor (MPC) is an open-source Java based modeling utility, built upon JSim's Mathematical Modeling Language (MML) ( http://www.physiome.org/jsim/) that uses directives embedded in model code to construct larger, more complicated models quickly and with less error than manually combining models. A major obstacle in writing complex models for physiological processes is the large amount of time it takes to model the myriad processes taking place simultaneously in cells, tissues, and organs. MPC replaces this task with code-generating algorithms that take model code from several different existing models and produce model code for a new JSim model. This is particularly useful during multi-scale model development where many variants are to be configured and tested against data. MPC encodes and preserves information about how a model is built from its simpler model modules, allowing the researcher to quickly substitute or update modules for hypothesis testing. MPC is implemented in Java and requires JSim to use its output. MPC source code and documentation are available at http://www.physiome.org/software/MPC/.

## Introduction

Many attempts have been made to provide modular modeling for physiological applications ( Erson & Cavuşoğlu, 2012; Krause *et al.*, 2010; Mirschel *et al.*, 2009; Smith *et al.*, 2009). We describe our modeling utility as semi-automated modular programming construction. It is simple and not conceptually novel, but is easy to learn and use. For developing a series of models of increasing complexity, Modular Program Constructor (MPC) can serve well as the primary basis for coding new model components and for incorporating modules of previously developed modeling code. The perspective is to take a modular approach; this means that one builds from simple modeling elements initially and then use multi-modular constructs as modules in higher level models.

Modular model creation and construction rely, to varying degrees, on meta-data to assist in reusing and merging previous models into a new one. Antimony ( Smith *et al.*, 2009) is the simplest approach. It requires the user to be familiar with the model and just specify that you want to import it into the new model. It relies on the user to resolve discrepancies between models. SemanticSBML ( Krause *et al.*, 2010), SemGen ( Gennari *et al.*, 2011; Neal *et al.*, 2015), and Phy-Sim ( Erson & Cavuşoğlu, 2012) make use of standard semantic and ontological descriptions of a biological model to allow large models to be broken down easily, without much user guidance, into biologically meaningful components linked to their mathematical description. Semantic and ontological metadata assists the construction of new models by providing suggested connections or relationships between models. This approach requires the user to invest time in complete annotation of models with standardized meta-data. The payoff is models that can be constructed and merged together using biological rather than mathematical terms. ProMot ( Mirschel *et al.*, 2009) enforces an object-oriented approach to modeling (defining external interfaces for each object) and attempts to use network theory to describe biological systems through specifying elements and coupling elements. MPC relies on the user to modularize a model using directives to specify them. MPC then requires the user to specify how the new model makes use of the modules. MPC only imposes unit balance constraints indirectly, through the JSim MML compiler ( Butterworth *et al.*, 2014).

In MPC, a module can be any set of variable declarations, parameter declarations and mathematical equations that represent a process. This broad definition of a module has a broad variety of applications: from a simple first order enzyme reaction, to a complete model of coronary blood flow through heart muscle, which can then be incorporated into a yet larger systemic model.

MPC is based upon ModelBuilder, which used FORTRAN to parse code and define directives ( Raymond, 2008). MPC is now Java based and simpler to use. Some MPC built models include time-dependent two-dimensional spatial models in both Cartesian and cylindrical coordinates ( Raymond & Bassingthwaighte, 2011; Raymond & Bassingthwaighte, 2012), and whole organ models with heterogeneous flows, and substrate metabolism, including reconstructing Bassingthwaighte *et al.*, 1989 blood-tissue exchange model.

## Methods

### MPC implementation

MPC is a pre-compiler written in Java. It reads a text input file, parses the file for directives, and generates a text output file based on those directives. MPC is built upon the Mathematical Modeling Language (MML) of JSim ( http://www.physiome.org/jsim/) [ Butterworth *et al.*, 2014]. It has been designed to work with JSim's MML and currently requires JSim to run the model output file that MPC produces. Through JSim, the final constructed model can be exported into Systems Biology Markup Language (SBML, http://sbml.org/Main_Page) or CellML ( https://www.cellml.org/), and imported to other SBML or CellML supported simulation platforms ( Smith *et al.*, 2014). MPC currently is executed as a command line utility and requires the Java runtime environment ( https://java.com/).

MPC has three components:

1. *MML*, the mathematical modeling language of JSim, is a declarative language which is used to specify all the model equations, leaving the sequencing and solving of equations to the JSim compiler and simulator. MML declares parameters and variables (with units), defines algebraic equations, ordinary and partial differential equations with their associated initial and boundary conditions.
2. *Modules* are MML model code which are variable declarations, parameter declarations, or mathematical equations for a particular process, for example, flow along a capillary, diffusion within a region, a chemical reaction, transport across a membrane, or even a whole organ. These are archived, forming libraries of operational module code that can be publicly distributed (some are available at http://www.physiome.org/software/MPC/). This allows the user to generate multi-scale models with different sub-models to use in testing a hypothesis against data, i.e. validity testing. For example, there have been a variety of models developed to describe the transmembrane sodium pump, NaKATPase which uses ATP to pump sodium out of, and potassium into, the cell ( Chapman *et al.*, 1983; Demir *et al.*, 1994; Goldman, 1943; Hille 2001; Hodgkin & Huxley 1952; Lauger 1991; Winslow *et al.*, 1999). All of these models have the same essential external influences: the Na and K ion concentrations and the transmembrane electrical potential. Having a library of the MML code for the variant modules allows one to insert one's choice quickly into the template for the cell model. Changing combinations rapidly to match solutions with experimental results is invaluable for the early phases of developing alternative hypotheses.
3. *Directives*, the third component, comprises the set of instructions used by the MPC model utility to select processes and gather the code from existing modules, renaming parameters and variables to reflect the new purposes for which they will function, and automatically combining the mathematical structures into new structures. The directives control the identification, fetching and relabeling of variables and parameters, and the assembly and recombination of model code into new equations. All MPC directives start with '//%' for identification by the MPC parsing algorithm.

### Selecting and arranging components using directives – A simple example

The MPC input file guides the construction of a model made of previously existing model modules. It combines MML with “directives” embedded as comments and uses code from other JSim model files that have been annotated so that they can be read by MPC, yet without interfering with their operability. MPC may also combine models with other models or with modules of preconstructed code from model code libraries. These modules are specified within a library with the START and END directive. A “library” with a few elementary operators from which we will build a model in our next step is illustrated below:

**CodeLibrary.mod:**



                        //------------------------- ODE DOMAINS
//%START      odeDomains
 // START...END directives used to specify a module. realDomain t s; t.min=0; t.max=16; t.delta = 0.1;
//%END        odeDomains
//------------------------- flowCalc
//%START    flowCalc
C:t = (F/V)*(Cin-C);
//%END  flowCalc
//------------------------- EXCHANGE CACULATIONS
//%START      exchangeCalc
C1:t = PS/V1*(C2-C1);    // Exchange between two compartments
C2:t = PS/V2*(C1-C2);
//%END        exchangeCalc
//------------------------- REACTION A->B
//%START      reactionCalc
real G = 5 ml/(g*min);   // Const reaction rate.
A:t = -G/V*A;
B:t = G/V*A;
//%END        reactionCalc
//------------------------- MM REACTION A->B
//%START      MMreactionCalc
real KmA =1.0 mM, VmaxA  =2 umol/(g*min); // MM constant and max velocity of rxn
real G(t) ml/(g*min);    // MM reaction rate
G = (VmaxA/(KmA+A));
A:t = -G*(A)/V;
B:t =  G*(A)/V;
//%END       MmreactionCalc
                    


**Figure 1.**
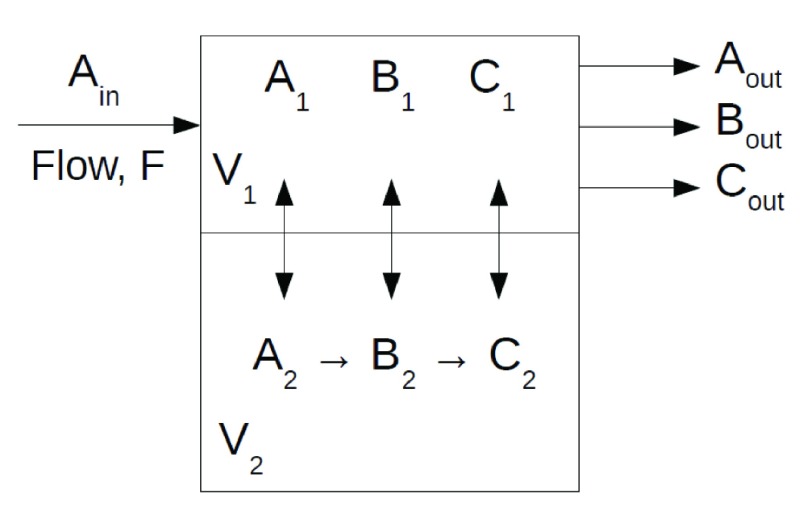
Two compartment, three species model (A, B, C) with volumes V _1_, V _2_, respectively. A _in_ is the concentration of A entering compartment 1 through which the flow is F. There is no flow in V _2_, but there are the reactions A-->B and B-->C. Passive exchange between compartments occurs for all three species.

In JSim's MML, the colon signifies the derivative: C:t means dC/dt. Comments are preceded by a double slash, ‘//’. Within MPC we can write MML code directly or import code from operational JSim models that have been annotated to identify components. An example is a three species (A, B, C), two compartment model with two reactions in compartment two ( Figure 1) with species concentrations described by ordinary differential equations (ODE). Species A enters, with flow F, a compartment with volume V1 and passive exchange between a second compartment with volume V2. In the second compartment, A reacts at rate GA2B to form B and B reacts to form C at rate GB2C, a Michaelis-Menten reaction.

The MPC file defines the domain, parameters, variables, and initial conditions first. Using the directives listed in ‘Example.mpc’, model code is extracted from the file ‘CodeLibrary.mod’ shown above. Values and variable names needing replacement throughout the final model are specified by the REPLACE directive along with the '%symbol%' placeholder. The use of the REPLACE, GET, COLLECT, INSERTSTART and INSERTEND directives are used in Example.mpc shown below:

**Example.mpc:**



                        //%REPLACE %CL% =("CodeLibrary.mod") // Library to get code from, replace all
				     // occurrences of %CL% with CodeLibrary.mod
//%REPLACE (%N%=("1","2"), %vol%=("0.05","0.05")) // Two compartments with volumes, replace
			      // all occurrences of %N% with 1,2 and %vol% with 0.05, 0.15
//%REPLACE (%AB%=("A","B","C") %PS3%=("6","5","4")) // 3 species, PS init values.
import nsrunit; unit conversion on; // Use cgs units for this model.
math example {         		    // model declaration
// INDEPENDENT VARIABLES
//%GET %CL% odeDomains()     // Get odeDomains section from CodeLibrary.mod
//%INSERTSTART a2bParmsVars  // Specify params and vars section
// PARAMETERS
real Flow = 1 ml/(g*min);    // Flow rate
real PS%AB%12 = %PS3% ml/(g*min);   // Conductances: PSA12,PSB12,PSC12
real V%N% = %vol% ml/g;	     // Volume of V1, V2
extern real %AB%in(t) mM;    // Inflowing concentrations
// DEPENDENT VARIABLES
real %AB%%N%(t) mM;          // A1,A2,B1,B2,C1,C2
// INITIAL CONDITIONS (IC's)
when(t=t.min)  %AB%%N%=0;    // Defines IC's for the ODEs
//%INSERTEND a2bParmsVars    // End params and var sec
//%INSERTSTART a2bCalc       // Specify calc section
// ODE CALCULATIONS
//%GET %CL% reactionCalc  ("A=A2","B=B2","V=V2","G=Ga2b")  // A->B reaction
//%GET %CL% MMreactionCalc ("A=B2","B=C2","V=V2","G=Gb2c", // B ->C MM reaction
//% "KmA=KmB2","VmaxA = VmaxB2", "KmA = KmB2")             // B ->C MM reaction continued
//%GET %CL% flowCalc ("Cin=%AB%in","C=%AB%1","V=V1","F=Flow","D=D%AB%1")
//%GET %CL% exchangeCalc ("C1=%AB%1","PS=PS%AB%12","C2=%AB%2")
//%COLLECT("%AB%%N%:t")      //Group all ODE calculations for a species together
//%INSERTEND a2bCalc }  // curly bracket ends model
                    


The GET directive warrants further explanation: it identifies a model code library file and module name within the library to insert into the model, and changes old names (names of parameters and variables in the module) to new model names. From the example above, //%GET %CL% reactionCalc ( "A=A2","B=B2","V=V2","G=Ga2b") will get the module named 'reactionCalc' in file 'CodeLibrary.mod' and replace the variable names with the new model names ( "A=A2", etc).

**Figure 2.**
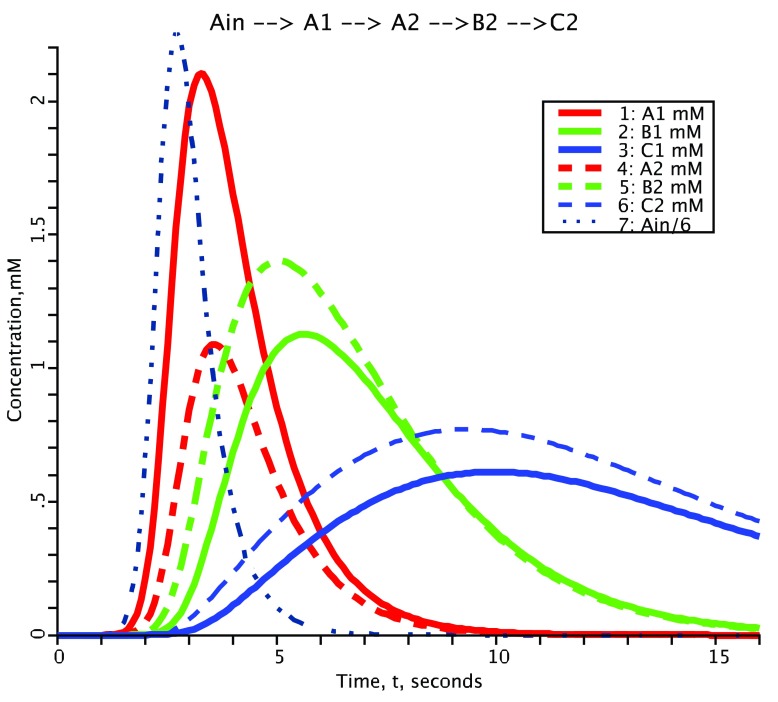
Solutions for the two compartment model generated from MPC. Species concentrations plotted as a function of time. Species A ( red), B ( green), C ( blue). Compartment 1: solid line, Compartment 2: dashed line. A _in_, the input function (black dot dashed line), is a lagged normal density function for species A. Values for the input function are: Area under curve = 20 mM•sec, relative dispersion, RD = 0.25, skewn: 1.3, mean time: 3 sec.. See ‘MPC Ouput-Example.mod’ for other parameter values and initial conditions.

 The MPC directives control the identification, fetching, relabeling of variables and parameters, and assembling and recombining code into new equations. The directives extract equations from files, changing the names of the module variables to application specific names and assemble the code into combined equations. The model code resulting from these instructions provides a complete program (Example.mod); in the following MPC output file (example.mod) some redundant comments have been removed, other explanatory comments have been added. The MPC generated program is ready to use with no further intervention on the part of the user except to adjust parameters or the solution time step length, and to set up graphics in JSim to display solutions, as shown in Figure 2.

**MPC Output - Example.mod:**



                        import nsrunit; unit conversion on;   // Use cgs units
math example {                        // model declaration
// INDEPENDENT VARIABLES
realDomain t s; t.min=0; t.max=16; t.delta = 0.1;
//%START a2bParmsVars       // Specify parameters and variables sect.
// PARAMETERS
real Flow = 1 ml/(g*min);   // Flow rate
real PSA12 = 6 ml/(g*min);  // Conductance
real PSB12 = 5 ml/(g*min);  // Conductance
real PSC12 = 4 ml/(g*min);  // Conductance
real V1 = 0.05 ml/g;        // Volume
real V2 = 0.05 ml/g;        // Volume
extern real Ain(t) mM;    // Inflow concentration of solute A
extern real Bin(t) mM;    // Inflow concentration of B, set to zero
extern real Cin(t) mM;    // Inflow concentration of C, set to zero
// DEPENDENT VARIABLES
real A1(t) mM; real B1(t) mM; real C1(t) mM; // Concentrations in V1
real A2(t) mM; real B2(t) mM; real C2(t) mM; // Concentrations in V2
// INITIAL CONDITIONS (IC's)
when(t=t.min)  A1=0;
when(t=t.min)  A2=0;
when(t=t.min)  B1=0;
when(t=t.min)  B2=0;
when(t=t.min)  C1=0;
when(t=t.min)  C2=0;
//%END a2bParmsVars    // End parameters and variables section
//%START a2bCalc       // Specify calculations section
real Ga2b = 5 ml/(g*min);   // A ->B First order reaction rate.
real KmB2 =1.0 mM, VmaxB2 =2 umol/(g*min);// Michaelis const; max velocity of rxn
real Gb2c(t) ml/(g*min);                  // B ->C Michaelis Menten reaction rate
Gb2c = (VmaxB2/(KmB2+B2));
// ODE CALCULATIONS
A2:t = -Ga2b/V2*A2 +PSA12/V2*(A1-A2);
B2:t = Ga2b/V2*A2 -Gb2c*(B2)/V2 +PSB12/V2*(B1-B2);
C2:t = Gb2c*(B2)/V2 +PSC12/V2*(C1-C2);
A1:t = (Flow/V1)*(Ain-A1) +PSA12/V1*(A2-A1);
B1:t = (Flow/V1)*(Bin-B1) +PSB12/V1*(B2-B1);
C1:t = (Flow/V1)*(Cin-C1) +PSC12/V1*(C2-C1);
// %END a2bCalc
}   // curly bracket ends model
                    


The process above is hardly worthwhile for small models but is highly efficient for larger models where flexibility in structure is desired. In the example above, converting the ODEs to PDEs requires a three line change. Addition of a new PDE e.g. for red blood cells in a capillary, takes four lines. For a five species, three region model, a three line change generates a 15 PDE model.

The small set of directives builds complex models from simpler model modules. MPC allows one to reliably reuse existing models in larger, multi-scale models. MPC encodes and preserves information about how a complex model is built from its modules allowing quick substitution of modules. The amount of actual code a user needs to write is reduced, especially for more complicated models. In MPC we have generated a full organ model with heterogeneity of flow, competitive transporters on the cell membranes, and reactions for multiple species ( Bassingthwaighte *et al.*, 2012) e.g. for adenosine processing in the heart. It is a 7-path, three region model that involves five species (adenosine, inosine, hypoxanthine, xanthine, and uric acid) in a sequential reaction chain. The model contains over 100 PDEs for convection, diffusion, and reactions. Please see more detailed examples in the ‘Data Availability’ and ‘Software Availability’ sections below.

## Discussion

A prerequisite to using MPC is semantic consistency throughout the libraries and modules. Automated systems using ontologies will help craft models ( Gennari *et al.*, 2011), but the great efficiency of MPC for model construction begins to show when there are many model modules as in biochemical networks and circulatory or airway models. The VVUQ process ( Johnstone *et al.*, 2016) provides key steps toward reproducibility (VVUQ = Verification, Validation, Uncertainty Quantification, the latter defining predictive accuracy). Though an MPC-generated model is checked for syntax and unit balance through JSim, further verification is required: analytical solutions can be written into the code to match specific limiting cases, but otherwise one depends on testing for mass, charge, or energy balances. Validation requires testing against data, independent of the construction method; model solutions should not be in contradiction to the data. Quantification of the uncertainty is needed for making predictions from the model: UQ includes uncertainty in parameters, handled by JSim's Monte Carlo analysis, and in inputs/environment and model structure. Structural uncertainty, a major challenge, defines a major role for MPC: inserting different choices from amongst similar but differently functioning modules, into a large, multi-modular model, and solving the system many times with the variant constituents illustrating uncertainty in the projected outcomes.

## Summary

A limited set of directives in MPC, our Modular Program Constructor, allows us to build complex models from small models of simple physiological processes. MPC encodes and preserves information about how a complex model is built from its simpler model modules allowing the researcher to quickly substitute or update modules to validate a hypothesis. The amount of actual model code a user needs to write is reduced, especially for more complicated models.

Future updates will improve collection and insertion of model code, better identify external model module 'connections' for easier incorporation into larger models, and more intelligent reconciliation of similar code between modules. The long-term strategy is to integrate MPC within JSim allowing the user to take advantage of JSim's MML compiler and graphical user interface to quickly merge code with less user intervention.

## Software availability

### Software access

The Java code for MPC, the examples presented here, some more detailed examples, and instructions are available at http://www.physiome.org/software/MPC/.

### Source code as at the time of publication

https://github.com/F1000Research/MPC/releases/tag/v1.0

### Archived source code as at the time of publication

http://dx.doi.org/10.5281/zenodo.34208

### Software license

MPC is released under a 3-clause ‘revised’ BSD license:

Copyright (C) 1999–2015 University of Washington

Developed by the National Simulation Resource

Department of Bioengineering, Box 355061

University of Washington, Seattle, WA 98195-5061.

Dr. J. B. Bassingthwaighte, Director

Redistribution and use in source and binary forms, with or without modification, are permitted provided that the following conditions are met:

* Redistributions of source code must retain the above copyright notice, this list of conditions and the following disclaimer.

* Redistributions in binary form must reproduce the above copyright notice, this list of conditions and the following disclaimer in the documentation and/or other materials provided with the distribution.

* Neither the name of the University of Washington nor the names of its contributors may be used to endorse or promote products derived from this software without specific prior written permission.

## Data availability

The data referenced by this article are under copyright with the following copyright statement: Copyright: © 2016 Jardine B et al.

The two compartment MPC built model, demonstrated here, is available at www.physiome.org (TwoCompExampMPC, Model # 0345). As it is an ODE model it could be translated to SBML or CellML, allowing researchers whose simulation systems support one of these markup languages to run this model. However, for this presentation we have provided only the MPC annotation in order to retain its simplicity.

MPC generated models for review at www.physiome.org are:

• Two compartment MPC built model demonstrated here ( http://physiome.org/jsim/models/webmodel/NSR/TwoCompExampMPC/).
• Concentration profiles in capillary and tissue when exchange is diffusion-limited ( http://www.physiome.org/jsim/models/webmodel/NSR/DiffusionLimitedProfiles/).
• ODE model of actin polymerization and depolymerization with tracking of bound nucleotide ( http://www.physiome.org/jsim/models/webmodel/NSR/ActinCycle1/).
• Multiple tracer dilution estimates of D- and 2-deoxy-D-glucose uptake by the heart ( http://www.physiome.org/jsim/models/webmodel/NSR/Kuikka1986BTEX30MP/).
