# Supplementary material for: Semi-automated Modular Program Constructor for physiological modeling: Building cell and organ models
Source: F1000Res. 2016 Jun 16;4:1461. Originally published 2015 Dec 16. [Version 3] doi: 10.12688/f1000research.7476.3 (PMC5488124; doi:10.12688/f1000research.7476.3)
Supplement: Peer review history

# Peer review: Referee response for version 3

**DOI:** 10.5256/f1000research.9641.r14422

**Published:** 2016-08-01

**Authors:** Dagmar Waltemath

**Affiliations:** 1 Department of Systems Biology and Bioinformatics, University of Rostock, Rostock, Germany

Thank you for the latest revision. Please consider changing the last sentence of the first paragraph in the introduction to: "and then use" --> "and then uses".

I have read this submission. I believe that I have an appropriate level of expertise to confirm that it is of an acceptable scientific standard.

# Peer review: Referee response for version 2

**DOI:** 10.5256/f1000research.9029.r13212

**Published:** 2016-05-19

**Authors:** Vanessa Díaz-Zuccarini

**Affiliations:** 1 UCL Mechanical Engineering , Multiscale Cardiovascular Engineering Group, University College London, London, UK

I think after the previous corrections the article is much more readable. It would be extremely helpful if the authors could update the manuscript with hyperlinks to concrete databases/examples and to provide a concrete example to illustrate their point about 'The amount of actual code a user needs to write is reduced, especially for more complicated models'.

 I strongly believe one of the roadblocks to a more widespread use of markup languages and the reusability of code is the perceived challenge by modellers/users of the task(s) involved into make their models 'shareable' and 'reusable'. A contrast between 2 examples or simply a better description of the effort it would concretely entail, would make their case much clearer.

 This isn't absolutely necessary for the article but since the authors are trying to make a point and for other researchers to use the tools they have developed and use for their own research, I believe this would be useful.

I have read this submission. I believe that I have an appropriate level of expertise to confirm that it is of an acceptable scientific standard.

# Response

**Published:** 2016-06-16

**Authors:** Bartholomew Jardine

**Affiliations:** University of Washigton, USA

*I think, after the previous corrections, the article is much more readable. It would be extremely helpful if the authors could update the manuscript with hyperlinks to concrete databases/examples and to provide a concrete example to illustrate their point about 'The amount of actual code a user needs to write is reduced, especially for more complicated models'.*

 Author: On page 7 in final paragraph before the “Discussion” section added the following sentence, with links:

 "Please see more detailed examples in the Data Availability and Software Availability sections below.”

 Listed in these sections are specific examples of models available on our website (physiome.org) created using MPC.   

*I strongly believe one of the roadblocks to a more widespread use of markup languages and the reusability of code is the perceived challenge by modellers/users of the task(s) involved into make their models 'shareable' and 'reusable'. A contrast between 2 examples or simply a better description of the effort it would concretely entail, would make their case much clearer.*

*This isn't absolutely necessary for the article but since the authors are trying to make a point and for other researchers to use the tools they have developed and use for their own research, I believe this would be useful.*

  Author: We added this paragraph in the Data availability section (page 8) to address this important point:

 “The two compartment MPC built model, demonstrated here, is available at www.physiome.org (TwoCompExampMPC, Model # 0345). As it is an ODE model it could be translated to SBML or CellML, allowing researchers whose simulation systems support one of these markup languages to run this model. However, for this presentation we have provided only the MPC annotation in order to retain its simplicity.”

# Peer review: Referee response for version 2

**DOI:** 10.5256/f1000research.9029.r13211

**Published:** 2016-04-18

**Authors:** Dagmar Waltemath

**Affiliations:** 1 Department of Systems Biology and Bioinformatics, University of Rostock, Rostock, Germany

Thank you for revising the manuscript. It was nice reading it. I only have a few minor things to note

1. Last sentence of first paragraph in the introduction: I suggest to generalise the sentence and cut "for multi-scale modeling". Also, I think it should be "uses" instead of "use".
2. Last sentence of first paragraph in Methods: "MPC currently is executed as command line utility" - I would write "as a command line utility".
3. in Methods, in the listing of the three components, I suggest to remove "not procedural like Fortran or Matlab", as it is hard to follow the sentence structure and the information not essential. Also, is MML really designed to solve equations? Or rather to provide the information to solve them?
4. in Methods, second point in the above listing: You mention several models ("For example, there have been a variety of models...") - would it be possible to link to these specific works, e.g., using citations?
5. Last sentence on page 3: "A "library" with a few..." -  I think it should read "in our next step" (instead of "in out next step")
6. End of first paragraph on page 4: "Species A enters, with flow F, a compartment... I had problems following the sentence, specifically because of "passive exchange between"... Can simplify the sentence structure or use two sentences instead?
7. First sentence  in Discussion: Should it be "Though an MPC-generated..." (instead of "a")?
8. same paragraph: "These are key steps towards reproducibility and the VVUQ process." I would have found it helpful to get a reference to the VVUQ process. Can you add one?
9. Grant information, last sentence: Please use a capital letter to start the sentence, and add two "the" - "The grants supported the whole group."

I have read this submission. I believe that I have an appropriate level of expertise to confirm that it is of an acceptable scientific standard.

# Response

**Published:** 2016-06-16

**Authors:** Bartholomew Jardine

**Affiliations:** University of Washigton, USA

Responses reflected in version 3 of manuscript.

1. *Last sentence of first paragraph in the introduction: I suggest to generalise the sentence and cut "for multi-scale modeling". Also, I think it should be "uses" instead of "use".*
  Author: Changed last two sentences of first paragraph (page 3) to generalize: “For developing a series of models of increasing complexity, Modular Program Constructor (MPC) can serve well as the primary basis for coding new model components and for incorporating modules of previously developed modeling code. The perspective is to take a modular approach; this means that one builds from simple modeling elements initially and then use multi-modular constructs as modules in higher level models."
2. *Last sentence of first paragraph in Methods: "MPC currently is executed as command line utility" - I would write "as a command line utility".*
   Author: Made change as suggested.
3. *in Methods, in the listing of the three components, I suggest to remove "not procedural like Fortran or Matlab", as it is hard to follow the sentence structure and the information not essential. Also, is MML really designed to solve equations? Or rather to provide the information to solve them?*
  Author: Updated and clarified sentence (page 3, Methods *1. MML*, paragraph): “MML, the mathematical modeling language of JSim, is a declarative language specifying all the model equations, leaving the sequencing and solving of equations to the JSim compiler and simulator.”
4. *in Methods, second point in the above listing: You mention several models ("For example, there have been a variety of models...") - would it be possible to link to these specific works, e.g., using citations?*
  Author: added the following references (page 3, Methods *2. Modules*, paragraph):
  - Chapman JB, Johnson EA, and Kootsey JM, Electrical and Biochemical Properties of an Enzyme Model of the Sodium Pump, Journal of Membrane Biology 74., 139-153, 1983
  - Demir S, Clark J, Murphey C, and Giles W, A mathematical model of a rabbit sinoatrial node cell.  Am. J. Physiol. Cell Physiol. 266: C832-C852, 1994
  - Goldman David E. Potential, impedance, and rectification in membranes, J Gen Physiol 27: 37-60, 1943.
  - Hille B. Ionic Channels of Excitable Membranes. 3rd Ed, Sunderland, MA, Sinauer, 2001.
  - Hodgkin AL and Huxley AF. A quantitative description of membrane current and its application to conduction and excitation in nerve. J Physiol. 500-544, 1952d.
  - Lauger P, Electrogenic Ion Pumps, 2nd Ed, Sinauer, Sunderland MA 1991
  - Winslow RL, Rice J, Jafri S, Marban E, and O'Rourke B, Mechanisms of altered excitation-contraction coupling in canine tachycardia-induced heart failure, II: model studies. Circ Res. 1999 Mar 19;84(5):571-86.    
5. *Last sentence on page 3: "A "library" with a few..." -  I think it should read "in our next step" (instead of "in out next step")*
  Author: Fixed typo (page 4, "Selecting and arranging..." paragraph): “A `library` with a few elementary operators from which we will build a model in our next step is illustrated below:”
6. *End of first paragraph on page 4: "Species A enters, with flow F, a compartment... I had problems following the sentence, specifically because of "passive exchange between"... Can simplify the sentence structure or use two sentences instead?*
  Author: Made into two sentences to make more readable (page 5, first paragraph):
  “Species A enters, with flow F, a compartment with volume V1 and passive exchange between a second compartment with volume V2. In the second compartment, A reacts at rate GA2B to form B and B reacts to form C at rate GB2C, a Michaelis-Menten reaction.”
7. *First sentence in Discussion: Should it be "Though an MPC-generated..." (instead of "a")?*
  Author: Agree. Corrected (page 8, first paragraph); an M is an aspirate.
8. *same paragraph: "These are key steps towards reproducibility and the VVUQ process." I would have found it helpful to get a reference to the VVUQ process. Can you add one?*
  Author: Rewrote the first paragraph (page 8) of the “Discussion” section and added the following reference:
  - R.H. Johnstone, et al., Uncertainty and variability in models of the cardiac action potential: Can we build trustworthy models?, J Mol Cell Cardiol (2015), http://dx.doi.org/10.1016/j.yjmcc.2015.11.018
  Updated paragraph:
  A prerequisite to using MPC is semantic consistency throughout the libraries and modules. Automated systems using ontologies will help craft models ( Gennari et al., 2011), but the great efficiency of MPC for model construction begins to show when there are many model modules as in biochemical networks and circulatory or airway models. The VVUQ process (Johnstone et al. 2015) provides key steps toward reproducibility (VVUQ = Verification, Validation, Uncertainty Quantification, the latter defining predictive accuracy). Though an MPC-generated model is checked for syntax and unit balance through JSim, further verification is required: analytical solutions can be written into the code to match specific limiting cases, but otherwise one depends on testing for mass, charge, or energy balances. Validation requires testing against data, independent of the construction method; model solutions should not be in contradiction to the data. Quantification of the uncertainty is needed for making predictions from the model: UQ includes uncertainty in parameters, handled by JSim's Monte Carlo analysis, and in inputs/environment and model structure. Structural uncertainty, a major challenge, defines a major role for MPC: inserting different choices from amongst similar but differently functioning modules, into a large, multi-modular model, and solving the system many times with the variant constituents illustrating uncertainty in the projected outcomes.
*I.  Grant information, last sentence: Please use a capital letter to start the sentence, and add two "the" - "The grants supported the whole group."*
  Author: updated sentence as suggested.

# Peer review: Referee response for version 1

**DOI:** 10.5256/f1000research.8055.r11594

**Published:** 2016-01-05

**Authors:** Dagmar Waltemath

**Affiliations:** 1 Department of Systems Biology and Bioinformatics, University of Rostock, Rostock, Germany

The manuscript at hand describes MPC, a tool that supports modelers in constructing complex models from smaller ones. MPC also keeps information about the single modules, making their exchange and further coupling even easier.

The manuscript provides several examples (on code and abstract level) of how to use MPC, but it does not give details on how the algorithm itself works.

My suggestions for improvements are mainly on the terminology used throughout the manuscript, and on the discussion of related work.

1. Unifying terms: In the abstract alone you speak about programs, utilities, code; about models, processes, model code and modules. Maybe you could - not only in the abstract but throughout the manuscript - unify your wording a little bit more to make the text more comprehensive.
2. Related work: I missed a discussion of related systems, e.g. the model merge tool for SBML, semanticSBML, or the semantic-based system (there was a new publication just recently ^1^). While you mention them in the beginning of your introduction, I did not see a discussion of these systems, and how they differ from your approach. I, as a reader, would be interested to know which system is best to use when. 

Furthermore, I have the following smaller comments:

1.  Page 2, Introduction: "The models include time-dependent..." -- Here it was not clear to me what you mean by "models".
2. Page 2, MPC implementation: "Through JSim, the final constructed model...." -- I understand here, that you can upload your constructed models from JSim into an open model repository, and then directly download them into other simulation platforms. I am not sure that it is as easy as this, particularly for BioModels there will be a curation process in between, and there is thus no immediate reuse. The way the sentence is written now, a reader may assume that models can directly and immediately be exchanged through these resources, which is in my opinion misleading.
3. Page 2, MPC implementation: "These are archived, forming libraries of operational code" -- I would be interested to know how you archive the modules, where, and how/if/to what degree they are accessible/reusable?
4. Figure 1: I would like to suggest using an SBGN-compliant notation for the toy model.
5. Summary: "MPC encodes and preserves..." -- This is an important information for the users, and I would like to suggest to add this information to the abstract.

I have read this submission. I believe that I have an appropriate level of expertise to confirm that it is of an acceptable scientific standard, however I have significant reservations, as outlined above.

## References

[1] NealMLCarlsonBEThompsonCTJamesRCKimKGTranKCrampinEJCookDLGennariJH: Semantics-Based Composition of Integrated Cardiomyocyte Models Motivated by Real-World Use Cases. *PLoS One*.2015;10(12) : 10.1371/journal.pone.0145621 e0145621 10.1371/journal.pone.0145621 26716837PMC4696653

# Response

**Published:** 2016-04-06

**Authors:** Bartholomew Jardine

**Affiliations:** University of Washigton, USA

Our responses to Referee Dagmar Waltemath's review:

1. *My suggestions for improvements are mainly on the terminology used throughout the manuscript, and on the discussion of related work. Unifying terms: In the abstract alone you speak about programs, utilities, code; about models, processes, model code and modules. Maybe you could - not only in the abstract but throughout the manuscript - unify your wording a little bit more to make the text more comprehensive.*
***Author Response:*** Yes, we updated the abstract and paper as a whole to try to use consistent and unifying wording when discussing model code, processes, modules, etc. These changes are most notable in the abstract and introduction.
2. *Related work: I missed a discussion of related systems, e.g. the model merge tool for SBML, semanticSBML, or the semantic-based system (there was a new publication just recently1). While you mention them in the beginning of your introduction, I did not see a discussion of these systems, and how they differ from your approach. I, as a reader, would be interested to know which system is best to use when.*
***Author Response:*** Added a paragraph in the Introduction that briefly discusses other tools in relation to MPC:
  "Modular model creation and construction rely, to varying degrees, on meta-data to assist in reusing and merging previous models into a new one. Antimony (Smith 2009) is the simplest approach. It requires the user to be familiar with the model and just specify that you want to import it into the new model. It relies on the user to resolve discrepancies between models. SemanticSBML(Krause 2010), SemGen (Genari 2011, Neal 2015), and Phy-Sim (Erson 2012)  make use of standard semantic and ontological descriptions of a biological model to allow large models to be broken down easily, without much user guidance, into biologically meaningful components linked to their mathematical description. Semantic and ontological metadata assists the construction of new models by providing suggested connections or relationships between models. This approach requires the user to invest time in complete annotation of models with standardized meta-data. The payoff is models that can be constructed and merged together using biological rather than mathematical terms. ProMot (Mirschel 2009) enforces an object-oriented approach to modeling (defining external interfaces for each object) and attempts to use network theory to describe biological systems through specifying elements and coupling elements (Mirschel 2009). MPC relies on the user to modularize a model using directives to specify them. MPC then requires the user to specify how the new model makes use of the modules. MPC only imposes unit balance constraints, indirectly, through the JSim MML compiler (Butterworth 2014)."

*Furthermore, I have the following smaller comments:*

1. *Page 2, Introduction: "The models include time-dependent..." -- Here it was not clear to me what you mean by "models".*
**Author Response:** Clarified sentence to make it clearer (Page 2, Introduction, 4th paragraph):
  Some MPC built models include time-dependent two-dimensional spatial models in both Cartesian and cylindrical coordinates (Raymond et al., 2011; 2012), requiring PDEs, and whole organ models with heterogeneous flows, and substrate metabolism, including reconstructing Bassingthwaighte et al., 1989 blood-tissue exchange model.
2. *Page 2, MPC implementation: "Through JSim, the final constructed model...." -- I understand here, that you can upload your constructed models from JSim into an open model repository, and then directly download them into other simulation platforms. I am not sure that it is as easy as this, particularly for BioModels there will be a curation process in between, and there is thus no immediate reuse. The way the sentence is written now, a reader may assume that models can directly and immediately be exchanged through these resources, which is in my opinion misleading.*
***Author Response:*** That sentence is confusing and not what we wanted to say. Changed to:
  Through JSim, the final constructed model can be exported into Systems Biology Markup Language (SBML, http://sbml.org/Main_Page) or CellML (https://www.cellml.org/), and imported to other SBML or CellML supported simulation platforms [Smith et al., 2014].
3. *Page 2, MPC implementation: "These are archived, forming libraries of operational code" -- I would be interested to know how you archive the modules, where, and how/if/to what degree they are accessible/reusable?*
***Author Response:*** Modules created and used by our team are currently available for download at physiome.org (http://physiome.org/software/MPC/) or search on term "mpc" (http://physiome.org/Models/modelDB/). At this time there are a very limited set of MPC modules available. Soon (May/June 2016) we will have individual MPC annotated modules accessible directly from our search page with all file dependencies listed and available for download as well as links to full JSim models that may use them. Contributions to our model repository are encouraged (Any modeling language accepted).
  Sentence inserted on page 2, in MPC implementation, paragraph 2, Modules: "These are archived, forming libraries of operational module code that can be publicly distributed (some are available at http://www.physiome.org/software/MPC/)."
4. *Figure 1: I would like to suggest using an SBGN-compliant notation for the toy model.*
***Author Response:*** Thank you for the suggestion, for this particular figure we would like to keep it as is, but since we are currently modeling cardiac metabolism at the sub-cellular level we will be adopting SBGN notation where possible. Arrowheads in figure are made smaller.
5. *Summary: "MPC encodes and preserves..." -- This is an important information for the users, and I would like to suggest to add this information to the abstract.*
***Author Response:*** Added this sentence to the abstract.
